# Stereological analyses of the whole human pancreas

**DOI:** 10.1038/srep34049

**Published:** 2016-09-23

**Authors:** Ananta Poudel, Jonas L. Fowler, Mark C. Zielinski, German Kilimnik, Manami Hara

**Affiliations:** 1 Department of Medicine, The University of Chicago, Chicago, Illinois 60637, USA

## Abstract

The large size of human tissues requires a practical stereological approach to perform a comprehensive analysis of the whole organ. We have developed a method to quantitatively analyze the whole human pancreas, as one of the challenging organs to study, in which endocrine cells form various sizes of islets that are scattered unevenly throughout the exocrine pancreas. Furthermore, the human pancreas possesses intrinsic characteristics of intra-individual variability, i.e. regional differences in endocrine cell/islet distribution, and marked inter-individual heterogeneity regardless of age, sex and disease conditions including obesity and diabetes. The method is built based on large-scale image capture, computer-assisted unbiased image analysis and quantification, and further mathematical analyses, using widely-used software such as Fiji/ImageJ and MATLAB. The present study includes detailed protocols of every procedure as well as all the custom-written computer scripts, which can be modified according to specific experimental plans and specimens of interest.

Insulin-secreting pancreatic beta-cells play a pivotal role in maintaining the glucose homeostasis. Regulation of beta-cell mass is an essential matter to understand the pathophysiology of diabetes. Recent studies have also demonstrated possible roles of other pancreatic cells in the pathogenesis of diabetes; glucagon-secreting alpha-cells^1^,^2^,^3^,^4^,^5^,^6^,^7^, somatostatin-secreting delta-cells^8^ and exocrine pancreatic tissues^9^,^10^. While a number of studies have shown marked species differences between mice and humans from gene expression to islet architecture^11^,^12^,^13^,^14^,^15^,^16^,^17^, human studies are still limited. We have shown the intrinsic regional variability in endocrine cell mass among head, body and tail regions of human pancreas^18^,^19^,^20^. Islet architecture is size-dependent in adults, where alpha- and delta-cell ratios to beta-cells increase in large islets, with more intermingled islet cells in structure^21^. The large size of the human pancreas technically hampers a comprehensive analysis of the whole organ, requiring a stereological approach. Currently, most of the studies examined a limited number of tissue sections from a restricted region(s) or even randomly selected samples from the whole pancreas with no regard to regional information. Furthermore, the endocrine cell mass is often assessed by selecting “islet-rich areas” out of an entire tissue section. Together with the variability in sample collection, various assessment methods of endocrine cell mass used by many different investigators hinder the direct comparison among a wealth of published studies in the field.

In this study, we aim to provide a foundation that should enable such comparisons. Prior algorithms for automated morphological analyses include software called “Pancreas^++^” 22. It measures islet and alpha-cell area and uses the difference to indirectly compute beta-cell area. Unfortunately, the software is currently not accessible. Commercial software such as Aperio GENIE (Leica Biosystems, Buffalo Grove, IL) and HALO (Indica Labs, Corrales, NM) are best suited for a quick analysis of a large number of samples with high-throughput image scanning such as in a clinical setting. Our method is optimized for precision, particularly for the quantification of various cell types in large organs using multi-fluorescent imaging, which has not been demonstrated before. As opposed to prior algorithms, here we provide the custom scripts that we use to the scientific community, which can be further modified as needed. We have developed a stereological method for quantitative analyses of the whole human pancreas, with large-scale computer-assisted unbiased quantification. Following the validation of the method, we have extensively performed various simulations to estimate a range of deviations that may result in from the applications of different criteria and methods that are commonly used.

## Methods

### Human pancreas specimens

Human pancreata were generously provided by the Gift of Hope Organ Procurement Organization in Chicago and the nPOD. The use of deidentified human tissues in the study was approved by the Institutional Review Board at the University of Chicago.

### Microscope and computing platforms

Microscopic images were taken with an Olympus IX8 DSU spinning disk confocal microscope (Melville, NY) with imaging software StereoInvestigator (MicroBrightField, Williston, VT). Image processing and analyses were carried out using custom-written scripts for Fiji/ImageJ (http://rsbweb.nih.gov/ij/). MATLAB (MathWorks, Natick, MA) and Mathematica (Wolfram Research, Champaign, IL) were used for mathematical analyses.

### Immunohistochemistry

The pancreas was divided into consecutive tissue blocks (~5 mm) with alternating collection between paraffin-embedding and snap frozen^18^. In this study, paraffin-embedded sections (5 μm) were stained with the following primary antibodies (all 1:500): polyclonal guinea pig anti-porcine insulin (DAKO, Carpinteria, CA), mouse monoclonal anti-human glucagon (Sigma-Aldrich, St. Louis, MO), polyclonal goat anti-somatostatin (Santa Cruz, Santa Cruz, CA), polyclonal goat anti-human PP (DAKO), and DAPI (Invitrogen, Carlsbad, CA). The primary antibodies were detected using a combination of DyLight 488, 549, and 649-conjugated secondary antibodies (1:200, Jackson ImmunoResearch Laboratory, West Grove, PA).

## Results

### Large-scale image capture and computer-assisted semi-automated analysis of the whole tissue section

**Figure 1:**
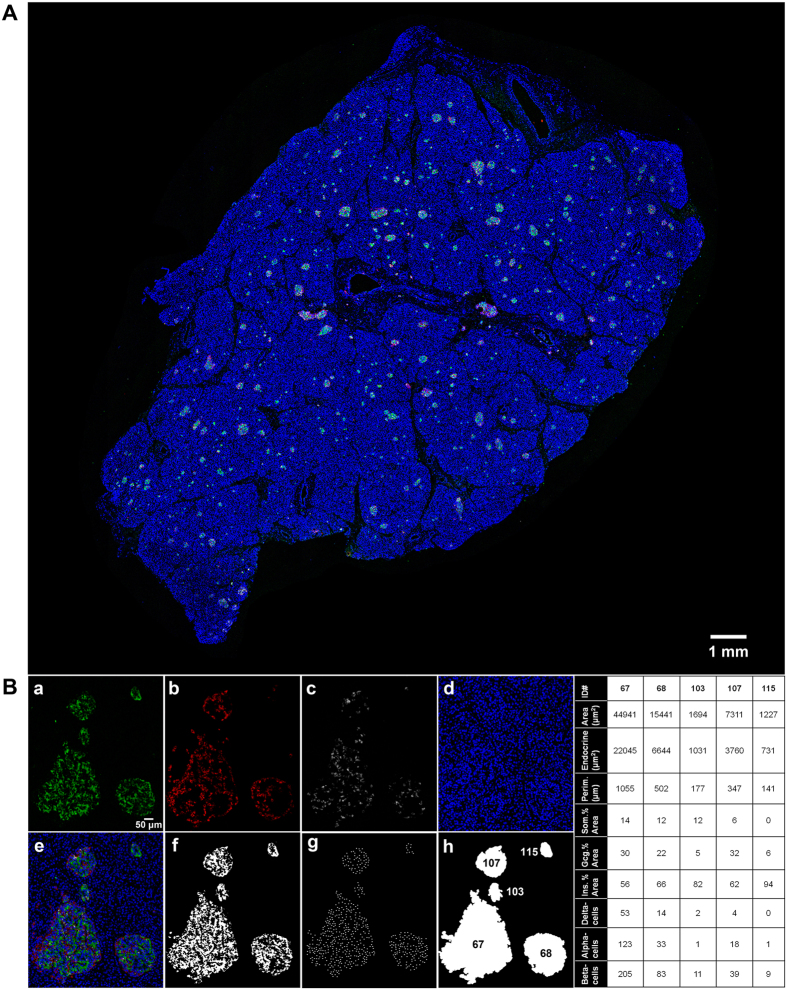
Computer-assisted large-scale image analysis. (**A**) Virtual slice view of a human pancreatic section (female, 2.2-y old, non-diabetic; nPOD #6107 Tail 02) immunostained for insulin (green), glucagon (red), somatostatin (white) and nuclei (blue). A series of contiguous optical panels of a specimen is collected and merged into a single image montage. A composite is made by merging four overlapping virtual slice images. (**B**) Views of each channel showing cellular composition of an islet cluster from the virtual slice in (**A**). (**a)** Beta-cells. (**b)** Alpha-cells. (**c)** Delta-cells. (**d)** Nuclei. (**e)** A composite of all three endocrine cells and nuclei. (**f)** Total endocrine cell area shown as a converted 8-bit mask after automatic thresholding. (**g)** Nuclei of endocrine cells. **(h)** Total islet area that includes unstained fractions such as intraislet capillary. Note that each islet including small clusters is designated with an identification number. Table summarizing data obtained through the computer-assisted large-scale analysis.

In order to describe our method in detail, a pancreas specimen obtained from the Network for Pancreatic Organ Donors with Diabetes (nPOD) was used (#6107 Tail 02, 2.2-yr old male, non-diabetic; Fig. 1). First, the entire tissue section is captured by a modified method of “virtual slice image capture” using a 10x objective^23^,^24^. One virtual slice image is typically composed of several hundreds of optical panels. Each virtual slice taken at multiple fluorescent channels is merged into one composite (Fig. 1A). The virtual slice image capture is shown in Supplementary Figure 1Aa.

The first step of image processing is “Rolling ball background subtraction” to remove large spatial variations of the background intensities, which is applied to all acquired images at 4 channels as a whole (i.e. no regional selection). In S.1A.b, the DAPI stained image after background subtraction is shown. To precisely quantitate endocrine cell mass, the most important step in our method is to determine the pancreas area, to which the endocrine cell mass is normalized. For the analysis of an entire tissue section, it is critical to consider the structural variability throughout the whole pancreas. For example, in human pancreas tissue sections, large vasculature areas can occupy >20% of the whole section. This will confound proper normalization with consistency and subsequent analyses of any types of comparisons such as between the affected versus the control as well as among each group. Here we manually contour “the pancreas area” to normalized to, excluding regions that do not contain endocrine cells/islets or exocrine cells and occupy >0.05% of a given section (S.1B), such as large blood vessels and ducts, periphery accumulated intra-pancreatic adipose tissue (which is often seen in obese subjects and the elderly), and empty space between lobes of the exocrine pancreas (which may be resulting from tissue preparation in some cases). The DAPI image is used for drawing a contour as shown in S.1A.c. A composite of 4 channels is used as a reference to ascertain endocrine cells/islets and “the pancreas area”. A contour can be drawn by using a computer mouse, or a graphics tablet that eases the labor as well as enhances the accuracy. After contouring, areas outside the contour are excluded (S.1A.d). This contour is then applied to other three channels to ensure that the same area to be analyzed as shown in S.1A.e–g. The set of four channels can be merged into a single RGB composite image with the three endocrine cell types and nuclei in different colors for visual clarity (S.1A.h). Images are prepared for analysis using the “Image processing macro” (S.2), and then the “Image analysis macro” (S.3) uses the processed images for quantification analysis. A macro is a custom-written script based on Fiji/Imagej functions and provides instructions for the quantification of interest in each application. The “Image processing macro” guides manually setting threshold values for all four channels, which will be saved as a text file. The processed images will be saved together in a single folder with specific file names. The “Image analysis macro” then uses the files in the folder for quantification of cellular composition (i.e., beta-, alpha- and delta-cell populations and nuclei; Fig. 1Ba–d). A composite of all three endocrine cells and nuclei is shown in Fig. 1Be. Total endocrine cell area is measured using converted 8-bit images (gray shades between black and white) after automatic thresholding (Fig. 1Bf). For later quantification, each endocrine cell nucleus area within islets is added (Fig. 1Bg). Total islet area that includes unstained fractions such as intraislet capillaries is measured by automated contouring of each islet structure (Fig. 1Bh). Each islet including small clusters is designated with an identification number so that specific information on individual islets can be obtained as summarized in the table.

The laboratory computer setup is critical, as large size multi-channel images obtained from the microscope require a high capacity transfer, storage and retrieval system. A wired Ethernet-connected network with server(s) and over 30 terabytes of storage capacity is recommended, as it facilitates easy transfer, retrieval and processing of images and data. A Linux based computer system operating on a server equipped with a multi-core processor, 32 GB of memory and extra memory swap space is optimal for running the ‘Image analysis macro’ on large image files. Fuji/ImageJ is compatible with Windows and Mac OS X, however, a Linux work environment is beneficial for batch processing, networking, scheduled routines, software redundant array of independent disks (RAID) for backups and access to open source command line tools useful for compressing and converting between large image file formats. In practice, images are uploaded to the server in the evening, allowing for automated analysis overnight.

### Measurements of spatial distribution of islets and each endocrine cell type within an islet

**Figure 2:**
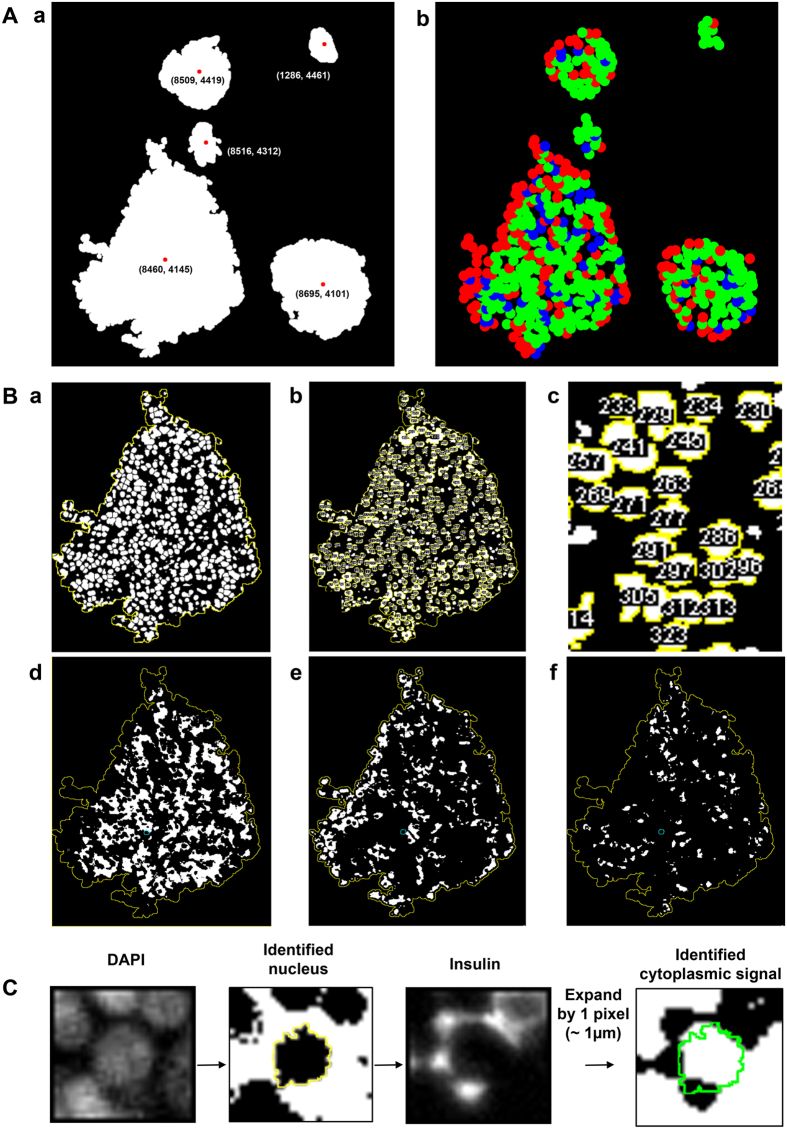
Measurements of spatial distribution of islets and each endocrine cell type within an islet. (**Aa**) Examples of measured centroids of islets and small clusters. (**b)** Reconstructed endocrine cell distribution within each islet (beta-cells in green, alpha-cells in red and delta-cells in blue). (**B**) Sequential analysis by the Image analysis macro. (**a)** Watershed segmentation applied to an 8-bit image converted from DAPI fluorescent signals. (**b)** Nuclei assigned with each specific identification number that corresponds to individual spatial coordinates of the nuclei. (**c)** Enlarged view showing a defined perimeter of each nucleus (a yellow line) with its own identification number. (**d)** Beta-cells. (**e)** Alpha-cells. (**f)** Delta-cells. (**C**) Automated identification of each cell type. A case of a single beta-cell is shown as an example. The perimeter of an identified nucleus (outlined as a yellow line) is expanded by 1 pixel (~1 µm) to detect the cytoplasmic region immediately surrounding the nucleus, which will determine its specific cell type.

**Figure 3:**
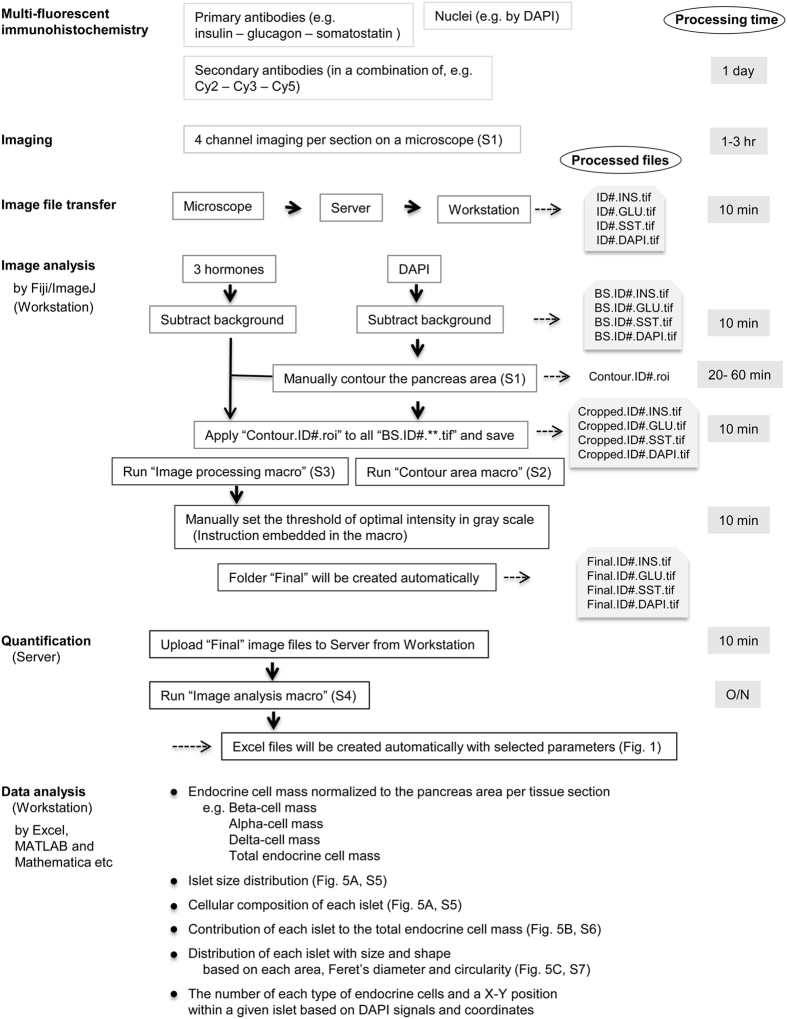
Schematic workflow. Starting with multi-fluorescent immunohistochemistry, 4-channel imaging is performed in a virtual-slice mode on a microscope. Captured images (1–2 GB each) are first transferred to Server (e.g. with storage capacity of 30 TB. Note that the Server serves for both image processing and storage of large data sets). Processed images are retrieved at Workstation for the subsequent image analysis. Our labeling scheme for processed images is shown on the right together with approximate processing time at each step. First, “Background subtraction” is applied to all channel images using Fiji/ImageJ. On the DAPI image, “the pancreas area” is manually contoured and is saved as a “.roi” file. The determined pancreas area is applied to images that have been background-subtracted (“BS.**.tif”) and the resulting image files are saved as “Cropped.**.tif”. Then by running the “Image processing macro”, manually set the threshold of optimal intensity in gray scale by following the instruction embedded in the macro. The macro automatically creates a set of image files that are ready for quantification. These files in the “Final” folder are manually uploaded to the Server for the quantification of various parameters. Run the “Image analysis macro” and Excel files will be automatically created that contain results with selected parameters. In the bottom box, examples of various analyses are listed with reference to each figure that are shown in the present study.

The spatial distribution of islets within a tissue section can be examined by measuring the centroid of each islet (Fig. 2Aa) using “Image analysis macro”. To further analyze the islet architecture, the center coordinates of each endocrine cell type within islets are measured. Reconstructed endocrine cell distribution within each islet is shown in Fig. 2Ab (beta-cells in green, alpha-cells in red and delta-cells in blue). The coordinate data are used to count the number of each endocrine cell type within islets, and for further analysis of cellular composition and geographic islet architecture (such as for mathematical modeling^25^,^26^,^27^,^28^,^29^). Sequential analysis by the Image analysis macro is outlined in Fig. 2B using one islet as an example. First, the DAPI fluorescent signals are converted to 8-bit images. The watershed segmentation is then applied to determine each nucleus boundary (Fig. 2Ba). After every nucleus has been identified, each one is assigned specific identification number that corresponds to individual spatial coordinates of the nuclei (Fig. 2Bb). An enlarged view in Fig. 2Bc shows a defined perimeter of each nucleus (a yellow line) with its own identification number. Next, all the identified nucleus perimeters are overlaid onto the three hormone signals, in order to determine which nucleus belongs to which specific cell type (Fig. 2Bd–f, beta-, alpha- and delta-cells, respectively). The automated identification of each cell type is illustrated in Fig. 2C at a single cell level using a beta-cell as an example. For each identified nucleus (outlined as a yellow line), the perimeter of the nucleus is expanded by 1 pixel (~1 μm) in order to detect the cytoplasmic region immediately surrounding the nucleus, which is used to determine the corresponding cell type. Here, this automated expansion could faultily include signals from other cell types due to the close proximity of each endocrine cell types within a given islet. Thus, the Image analysis macro is further designed to determine the most prevalent signals in order to distinguish a specific cell type (S.3). A video capturing the computer-assisted analysis is provided online (Video 1). A schematic overview of the workflow is shown in Fig. 3.

### Simulation to examine a possible sampling bias resulting from selecting islet-rich areas

To clarify the rationale of our approach, we ran a simulation analysis to compare to the widely-used method that multiple regions out of a single section are measured, and further the average of the triplicate (or more) of a given block of tissue is considered to represent the total endocrine cell area/mass.

**Figure 4:**
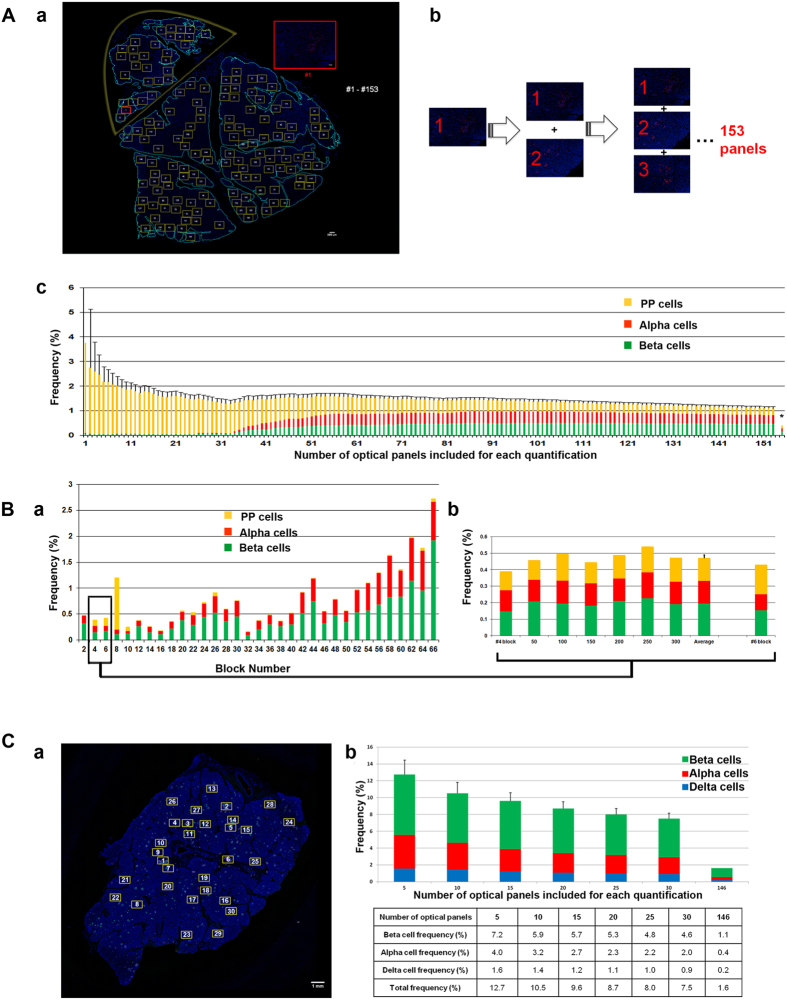
Selection of islet-rich areas in pancreas sections. (**A**) Simulation 1 using a pancreatic tissue section from the head region that contains PP-cell rich and poor area. (**a)** Pancreas area is contoured in light blue. PP-cell rich area is encircled in yellow. Inset: the first boxed area captured using a 10x objective shown in red. Optical panels were sequentially selected as enumerated in yellow boxes. (**b)** Selected panels were systematically added up to a total of 153 panels. (**c**) The cumulative averaged value of each quantification is plotted. An asterisk depicts the value obtained by our large-scale quantification. (**B**) Measurement of endocrine cell area on every 50^th^ section within a block. (**a)** Whole pancreas analysis of endocrine cell area (i.e. PP, beta and alpha-cells) from head, body to tail region (from left to right). (**b)** Block #4 was selected because it showed a similar endocrine cell mass to Block #6. The entire block of #4 was cut in 5 μm thickness, which yielded >300 sections. Total endocrine cell area was measured in every 50^th^ section by applying our large-scale analysis. (**C)** Simulation 2 using the nPOD specimen shown in Fig. 1. (**a)** To examine the commonly used selection of islet-rich areas, optical panels were sequentially selected up to 30 panels, as enumerated in yellow boxes. Note that each area of an optical panel is equivalent to that of using a 20x objective. (**b)** The range of overestimation resulting from the selection is shown compared to the large-scale analysis.

For the first simulation, we selected an extreme case that contains both PP-cell rich and poor areas in the head region of a human pancreas (Fig. 4A). Here, we aim to show how selection of specific areas leads to an inaccurate quantification. We started with the most PP-cell rich area (a red box in Fig. 4Aa) with the assumption that any investigator would look for a cluster of large endocrine cells/islets and try to capture as much as possible within an optical panel to obtain meaningful data. Starting with the most PP-cell rich area, other PP-cell rich panels were systematically added, eventually adding non-PP-cell rich areas declining in islet count until all islets were accounted for up to a total of 153 panels (optical panels chosen are in yellow and enumerated sequentially). Each panel was analyzed for endocrine cell area, and divided by the total pancreatic area to obtain percent endocrine cell area. The bar marked with the asterisk is the actual average endocrine cell area using our large-scale quantification method. These results suggest that the selection of islet-rich areas may overestimate endocrine cell mass by as much as 3 to 10-fold. This overestimation stems from insufficient normalization, where “the pancreas area” in our quantification consisted of 556 optical panels as opposed to the maximum of 153. Importantly, the resulting sampling bias will not be corrected by increasing the number of sections within a given block of a tissue, because the same sampling bias will be carried over to each section.

Next, we tested possible variability within one block of tissue (Fig. 4B). We cut an entire block of #4 in 5 μm thick, which yielded >300 sections. We examined every 50^th^ section by applying our method. The rationale for selecting the block #4 is that a single section from #4 block and that from #6 block gave similar values of each endocrine cell mass. These two sections were apart in ~1 cm in our preparation. Compared to the variability in the total endocrine cell area within the whole pancreas in the range of 0.15–3.0% (Fig. 4Ba), differences in endocrine cell mass in 6 sectioned examined within the block were in the range of 0.03–0.12% (Fig. 4Bb). Therefore, one section per block provides representative quantification, when the entire section is measured.

Using the nPOD specimen in the tail region (i.e. with no PP-cell rich area) with three major hormones staining (i.e. insulin, glucagon and somatostatin) shown in Fig. 1, we attempted to replicate a common method of selecting multiple regions out of a single section, by starting with the most islet-rich area (Fig. 4Ca). According to most applications seen in published papers, optical panels were sequentially selected up to 30 panels with each area equivalent to that using a 20x objective, as enumerated in yellow boxes. The average values of endocrine cell mass by selecting 5 to 30 optical panels are shown in Fig. 4Cb. Compared to the whole section analysis that takes 146 optical panels, the resulting overestimation was 4.5 to 8-fold.

### Comparison to the widely used point counting morphometry

**Figure 5:**
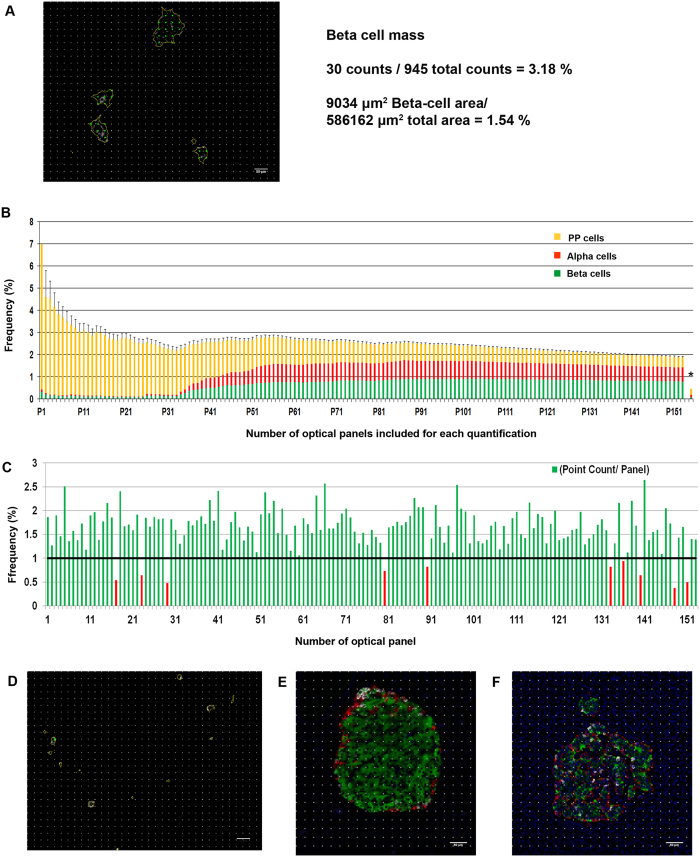
Comparison to the widely used point counting morphometry. **(A**) An example of comparison using a grayscale panel of insulin-positive beta-cells, overlaid with a regular square grid with vertices spaced at 25 µm. The extent of beta-cell mass classified is outlined in yellow. Vertices registered as positive are highlighted in green. (**B**) Simulation using the same pancreatic tissue section from the head region that contains PP-cell rich and poor area shown in Fig. 2A. (**C)** Comparison in each optical panel. Differences are normalized to the measurement of our method and shown as a ratio. Underestimated values are shown in red bars. (**D)** A representative view of panels that resulted in underestimation. (**E)** An example of quantification in mouse and human islets when point counting morphometry is applied.

Point counting morphometry is the most widely used method to quantify beta-cell mass in the pancreas to date. Figure 5A shows an example of comparison using a grayscale panel of insulin-positive beta-cells. Point count consists of a regular square grid with vertices spaced ~25 μm, shown as white dots in image overlay. Yellow outlines around beta-cells show the extent of beta-cell mass classified. Vertices registered as positive are highlighted in green. Total beta-cell area is measured as a ratio of positive to negative grid vertices (total 945 points). In this panel, 30 points were counted giving beta-cell mass of 3.18%, whereas it is quantified as 1.54% by our method.

Next, we carried out basically the same simulation described in Fig. 4A,B, but using the point counting morphometry. As a result, the range of overestimation markedly increased additively over that from the selection of islet rich areas in the range of 4 to 14-fold (Fig. 5B). Comparison in each optical panel shows that this overestimation occurs in most of them (Fig. 5C). Underestimation in some panels (red bars in Fig. 5C) occurs when a panel contains singly scattered endocrine cells, which often fall between points (Fig. 5D). The overall marked overestimation may be also due to the unique architecture of human islets compared to rodent islets that feature a beta-cell core. Figure 5E illustrates a possible degree of overestimation of point counting morphometry between mouse and human islets (30.4% vs 22.3% and 19.3% vs 7.4%, respectively).

### Islet size distribution and cellular composition

**Figure 6:**
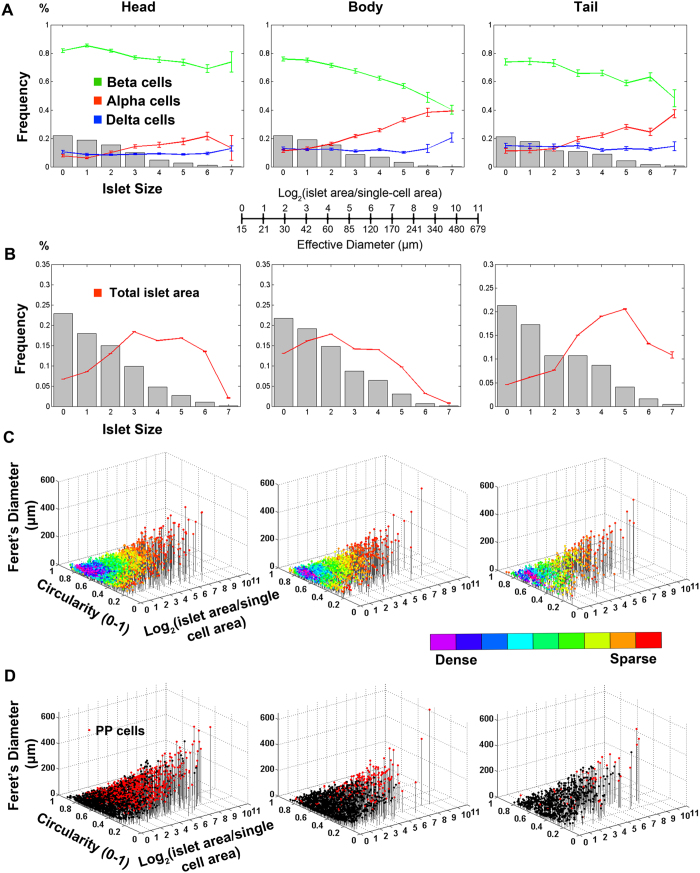
Islet size distribution, cellular composition. Specimens from head, body and tail region of nPOD #6107 were analyzed. (**A)** Frequency of islet size (gray bar) and ratios of beta-cells (green), alpha-cells (red) and delta-dells (blue) within islets are plotted against islet size; means ± SEM. Note that islet size is presented as a logarithmic scale considering the high number of small islets and the low number of large islets. In addition, islet area is divided by the single-cell area (178 μm^2 ^31) to make them as dimensionless values representing the number of cells in a given islet area. See the conversion between logarithmic islet area (logarithmic) and effective diameter (μm). (**B)** Fraction of islet size distribution (gray bar) and total islet area (red line). (**C)** 3D visualization of islet size and shape distribution. Each dot represents a single islet/cluster with reference to size (area) and shape (circularity and Feret’s diameter). The density of islets is color-coded from sparse to dense. (**D)** 3D scatter plots highlighting PP-containing islets.

Using a computing platform such as MATLAB or Mathematica, various targeted analyses can be performed based on large-scale data sets obtained through Fiji/Image J image analyses as described above. Islet sizes are in a wide range from a single endocrine cell to a large islet consisting of several thousand cells. In addition, smaller islets (including single cells and small clusters) are more frequent. Traditionally, such an analysis has been done by grouping islets as small, medium and large in size and the results are summarized in each category. Our aim is to capture a dynamic change of cellular composition throughout its wide islet size distribution (Fig. 6A). Here we have employed a logarithmic size scale for the histogram of islet sizes, because it gives fine bins for the high number of small islets and large bins for the low number of large islets. This provides not only sharp size categories of small islets but also statistically adequate islet counts at large size bins. In the head region (nPOD #6107, 2.2-yr old), beta-cells compose ~80% throughout the size distribution, whereas the fraction alpha-cells increases in the body and tail region.

Figure 6B shows the relative contribution of each bin of small to large islets to total endocrine cell area. The greater number of endocrine clusters and small islets (gray bars) does not markedly share the total area (plotted in a red line), but the fewer number of large islets mainly comprises the islet mass.

Additional parameters we measure are circularity and Feret’s diameter of each islet structure. Circularity reports the degree of roundness of a structure, where 1.0 corresponds to a perfect circle. Feret’s diameter is the longest distance within a structure. Circularity and Feret’s diameter are closely related that together depict a shape of a given structure^21^. With these parameters of area, Feret’s diameter and circularity, the entire distribution of endocrine cells and islets in the whole specimen can be visualized in a 3D scatter plot (Fig. 6C). The color-coding reports the density of a cluster with similar size and shape. The high number of singlets and small clusters of endocrine cells are densely clustered at the bottom of the 3D scatter plot. The low number of large islets is shown as singly scattered (i.e. red dots) with various sizes and shapes from spherical to elongated structure.

The 3D scatter plot shown in Fig. 6D is to visualize the regional distribution of a specific cell type, in this case PP-cell containing islets marked in red. Significant PP-cell segregation in the head region can be visually demonstrated in contrast to markedly less PP-cells in the body and tail region.

Custom-written scripts for these analyses are provided in S.2–7.

### Inter-individual heterogeneity in beta-cell/endocrine cell mass

**Figure 7:**
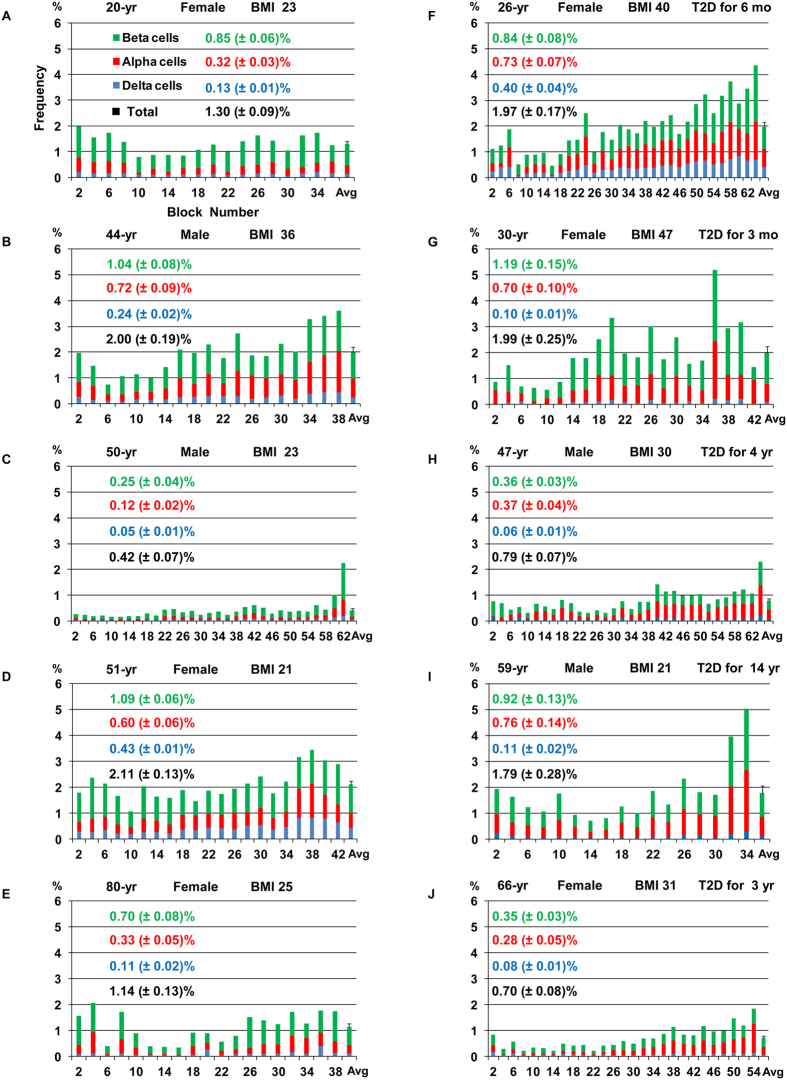
Whole pancreas analysis. **(A–E)** non-diabetic subjects. **(F–J)** patients with T2D. X-axis: Block number from head, body to tail region (from left to right).

Whole pancreas analysis has revealed marked intra-individual variability (i.e. regional differences), as well as inter-individual heterogeneity in beta-cell/islet mass. Figure 7 shows 10 cases of the whole pancreas analysis from head, body to tail region. All the graphs are plotted in the same scale for a better comparison. Overall, regional distribution of beta-cell/islet mass shows a gradual increase from head to tail region, although it fluctuates markedly. There appears no direct correlation regarding age and sex. Obese individuals, non-diabetic B and the most of patients with T2D except I, show no distinction from those with normal weight. Heterogeneity among patients with T2D, and further comparing to non-diabetic subjects, is striking.

### Possible bias in estimating the total endocrine cell mass in individuals by selecting a specific pancreatic tissue block out of the whole human pancreas

**Figure 8:**
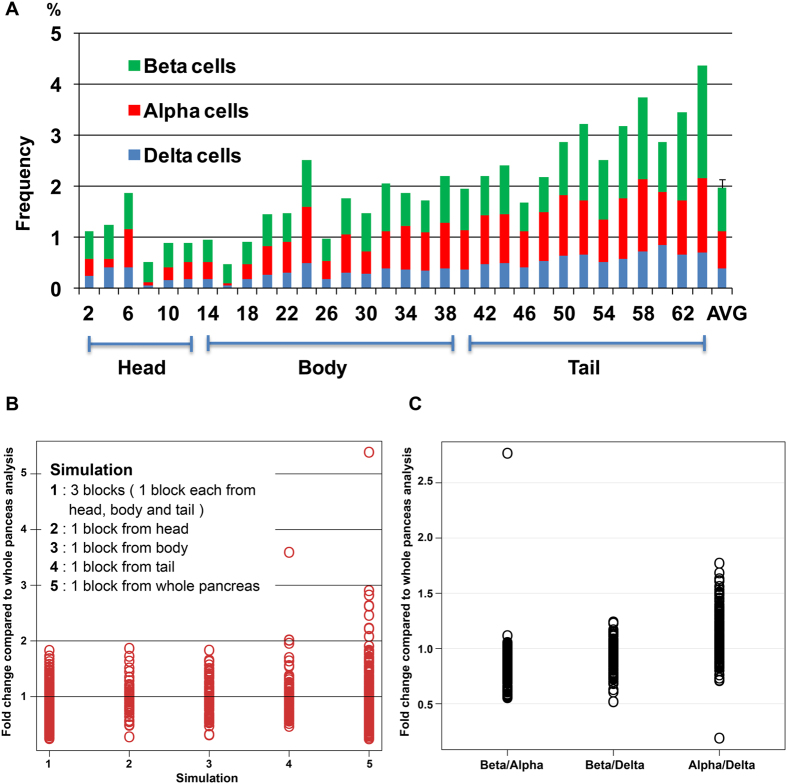
Simulation of tissue sampling. **(A)** Sampling scheme. The boundary of the head region was anatomically determined at the neck, and the remaining portion was divided in half for body and tail regions. (**B)** Fold changes in islet cell mass from 5 types of simulations compared to the whole pancreas analysis. (**C)** Fold changes in ratios of islet cells (i.e. beta/alpha, beta-delta and alpha/delta cells) from selecting 100 large islets compared to the whole pancreas analysis.

Many studies in the past examined a restricted region of the entire pancreas or tissues with no information about the regional origin. Here, we examined what ranges “estimated islet mass” can deviate depending on selection, using 10 cases shown in Fig. 8. The boundary of the head region was anatomically determined at the neck, and the remaining portion was divided in half for body and tail regions (e.g. Fig. 8A; Case F). Our simulation scheme was to cover every possible selection. Results of the simulated analysis of each individual case are summarized in Table S1. We then combined all these results from 10 cases and examined a range between the maximum and the minimum overestimation resulting from 5 different selection methods compared to the whole pancreas analysis (dot plots in Fig. 8B). Basically, any selection can result in ~2 fold underestimation or overestimation, even based on our whole pancreas tissue analysis. Note that the selection of “islet-rich” areas as we described in Figs 3 and 4, the overestimation can be multiplied ~5 to 10-folds.

With an emerging notion of non-beta-cell conversion to beta-cells^1^,^2^,^3^,^4^,^5^,^6^,^7^,^8^,^9^,^10^, possible changes of the beta:alpha:delta cell ratio is often measured by selecting a certain number of islets within a given tissue section. Here we systematically selected 100 islets starting with the largest one in each section/block in all the 10 cases, and compared to the whole pancreas tissue analysis (Fig. 8C). The average number of total islets per section in this study was >500, thus the largest 100 islets roughly comprise ~20% of the total islets per section. An overall tendency of underestimation of the beta/alpha cell ratio suggests an overestimation of the alpha-cell number, since the alpha-cell ratio within an islet increases with the size of islets in humans^21^. Note that this intrinsic size-dependent variability of human islets can contribute to an inaccurate representation of cell ratio, even when a reasonably large number of 100 islets is analyzed. As an extreme comparison, we also examined the alpha/delta cell ratio. When both populations are significantly smaller than that of beta-cells, the selection markedly affects the accuracy of measurements.

## Discussion

The stereological approach to perform a comprehensive analysis of the whole organ is widely applicable to various types of tissues from small to large animals. The most important concept in our computer-assisted methods is the accuracy, not to reduce the workload nor to accomplish quick analyses. The automated computer analyses are employed to exclude humane “selections” in order to ensure unbiased quantification. Selection of “islet rich areas” results in inappropriate normalization by excluding a large area of exocrine pancreas, which is prone to 5 to 10-fold overestimation of beta-cell/islet mass. Note that multiple sampling from a given block of tissue will not compensate this bias, but it will only be carried over. With large-scale image capture of an entire tissue section and subsequent computer-assisted image analysis, we have shown that one section per block sufficiently represents beta-cell/islet mass of a given tissue block.

The challenge in studying the human pancreas lies in the intra-individual variability, i.e. regional differences in endocrine cell mass from the head, body to tail region (in average ~1:1:2, respectively). It indicates that the regional information is critical in comparative studies. The inter-individual heterogeneity in beta-cell/islet mass is far more complex as shown in Fig. 7. In large cohort studies, a statistically significant difference may be observed, however, “the average value” is not likely useful to evaluate individual patients in clinical settings. On the other hand, in a simple comparison of two groups of “affected” versus “controls”, only a few number of “outliers” in the affected group can often shift the average value to the level of statistical significance, while the majority falls in a similar range with controls. Collectively, some studies may need to be reevaluated considering possible sampling bias that may stem from the intra-individual variability and the inter-individual heterogeneity.

In order to provide a baseline for reasonable as well as practical comparisons among the past and ongoing studies in the field, we examined ranges of deviation in “estimated islets mass” resulting from all the possible selections (Fig. 8). Overall, the deviation is ~2-fold, either overestimated or underestimated. Note that the quantification is based on our methods, where improper normalization can add up several-folds deviations. The interpretation of this 2-fold deviation, whether it is considerable or negligible, depends on a scale of differences observed in each comparative study. Similarly, we examined ranges of deviation in “estimated changes in islet cell ratios” (i.e. the beta:alpha:delta cell ratio) by systematically selecting 100 islets starting with the largest one. In average, 100 islets comprised ~20% of the total islets per section. Compared to the whole pancreas tissue analysis, the beta/alpha cell ratio was underestimated, which decrease in diabetic patients is supposed to “imply” an increase in alpha-cell mass^30^. This discrepancy should stem from the intrinsic characteristic of human islets that the ratio of alpha- to beta-cells increases in larger islets^21^.

For more general applications, based on large-scale data sets, various targeted mathematical analyses can be performed, including system-level mathematical modeling such as islet formation^25^, islet size distribution^26^, islet spatial distribution^27^, islet organization^28^ and beta-cell connectivity^29^.

Multi-fluorescent immunohistochemistry combined with large-scale image capture and analyses provides accurate quantification of each cell type as well as spatial distributions of several cell types within the same tissue section. Currently, we can perform up to 4-channel imaging with no overlaps between channels (See *Microscope* in the Methods). In addition to nucleus staining (e.g. DAPI), which is important for identification of specific cell types, cell number counting and coordinate measurement, various combinations of three cell types can be chosen. The present study provides a practical approach for performing comprehensive histological analyses on human and animal organs regardless of size.

## Additional Information

**How to cite this article**: Poudel, A. *et al*. Stereological analyses of the whole human pancreas. *Sci. Rep*. **6**, 34049; doi: 10.1038/srep34049 (2016).

## Supplementary Material

Supplementary Information

Supplementary Video S1
