# Supplementary material for: Stereological analyses of the whole human pancreas
Source: Sci Rep. 2016 Sep 23;6:34049. doi: 10.1038/srep34049 (PMC5034323; doi:10.1038/srep34049)
Supplement: Supplementary Information [file srep34049-s1.pdf]

Stereological analyses of the whole human pancreas

Ananta Poudel, Jonas L. Fowler, Mark C. Zielinski[1], German Kilimnik and Manami Hara

Department of Medicine, The University of Chicago, Chicago, Illinois 60637

[1]Current address: Department of Psychology, Brandeis University, Waltham, MA 02453

Correspondence to: Manami Hara, D.D.S., Ph.D., Department of Medicine,

The University of Chicago, 5841 South Maryland Avenue, MC1027, Chicago, IL 60637.

Tel: (773) 702-3727. Fax: (773) 834-0486. Email: mhara@uchicago.edu.

Figure Legends for Supplementary Information

S1. Large scale virtual slice image capture and manual contouring of the pancreas area for normalization. A. Image capture and pancreas area contour. **a.** A whole section image capture by sequential tiling of optical panels. Manually-set focus points are indicated in red. Note that shown here is a screen-capture of the microscope computer for an inverted microscope so that the right-left orientation is mirrored to the subsequent images for analysis. **b.** DAPI stained image after background subtraction. **c.** Contoured pancreas area in yellow outline. **d.** Selected pancreas area. **e-g.** Endocrine cells in the selected pancreas area (insulin, glucagon and somatostatin, respectively. **h.** A composite of all 4 channels. **B.** Percent area of non-pancreatic regions within the whole tissue listed from the largest to smallest (>0.05%) that can affect proper normalization.

Supplementary Video 1. Video with narration showing automated analysis using the "Image analysis macro".

0.00-0.05 First, the macro opens the four channel images in Fiji/ImageJ and applies the set threshold to all channels.

0.06-0.17 Insulin, glucagon and somatostatin are merged together, and islet structures are identified in the entire section, shown by the yellow outlines as regions of interests (ROIs).

0.17-0.22 In the given tissue section, 1,034 islets have been identified.

0.23-0.28 All islets in the section are assigned an ID number, and several islet parameters are measured.

0.29-0.32	Next, individual islets are analyzed.
0.33-0.46	Within a given islet, the macro applies watershed segmentation to separate nuclei. For each nucleus, the macro will identify which of the three hormone signals surrounding the nucleus is most prevalent in order to identify the specific cell type.
0.47-0.52	Results of the analysis are automatically saved in Excel files in the corresponding folders.

S2. **Contour area macro**. Fiji/ImageJ script calculates an area of the contour using the appropriate specified image scale.

S3. **Image processing macro.** Fiji/ImageJ guides user to set threshold values for all 4 channel images. Threshold values are automatically saved in a text file within a final folder that includes all 4 channel images for further analysis.

S4. Image analysis macro. Bash script, written using Fiji/ImageJ, analyzes 4 channel images by recording measurements of islet parameters and identifying specific cell types within islets.

S5. Script for plotting islet size distribution and cellular composition. Matlab script generates a plot showing cellular composition in relation to islet size distribution.

S6. Script for plotting relative contribution of islet sizes to the total endocrine cell area. Matlab script generates a plot showing a total endocrine cell area in relation to islet size distribution.

S7. Script for 3D scatter plots. Matlab script generates a 3D scatter plot visualizing individual islets in relation to shape (Feret's diameter and circularity) and islet size distribution. Density of islets is shown as a heat map between blue and orange representing dense (highly populated) to sparse (single islets), respectively.

Figure S1

A

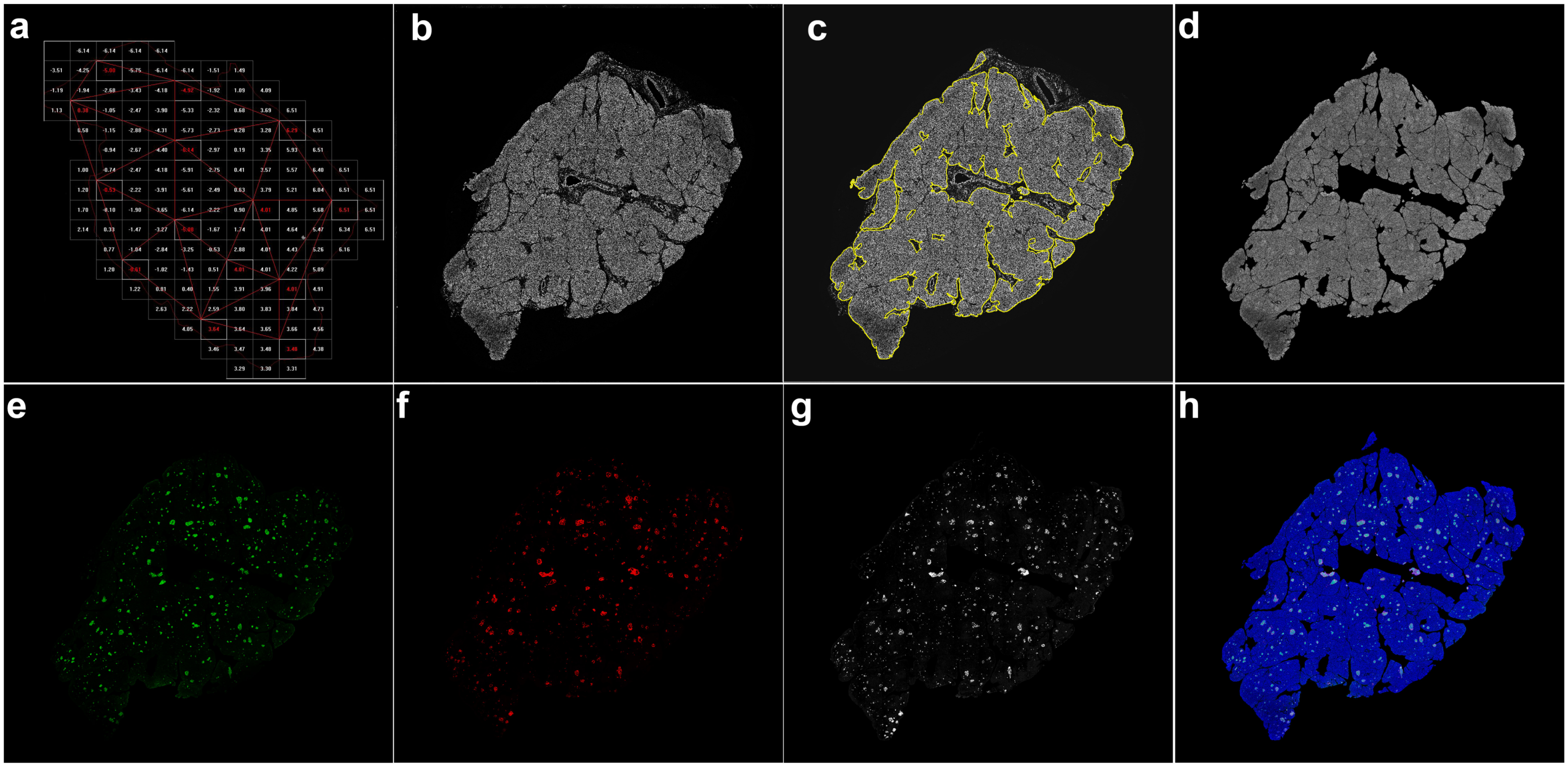

B

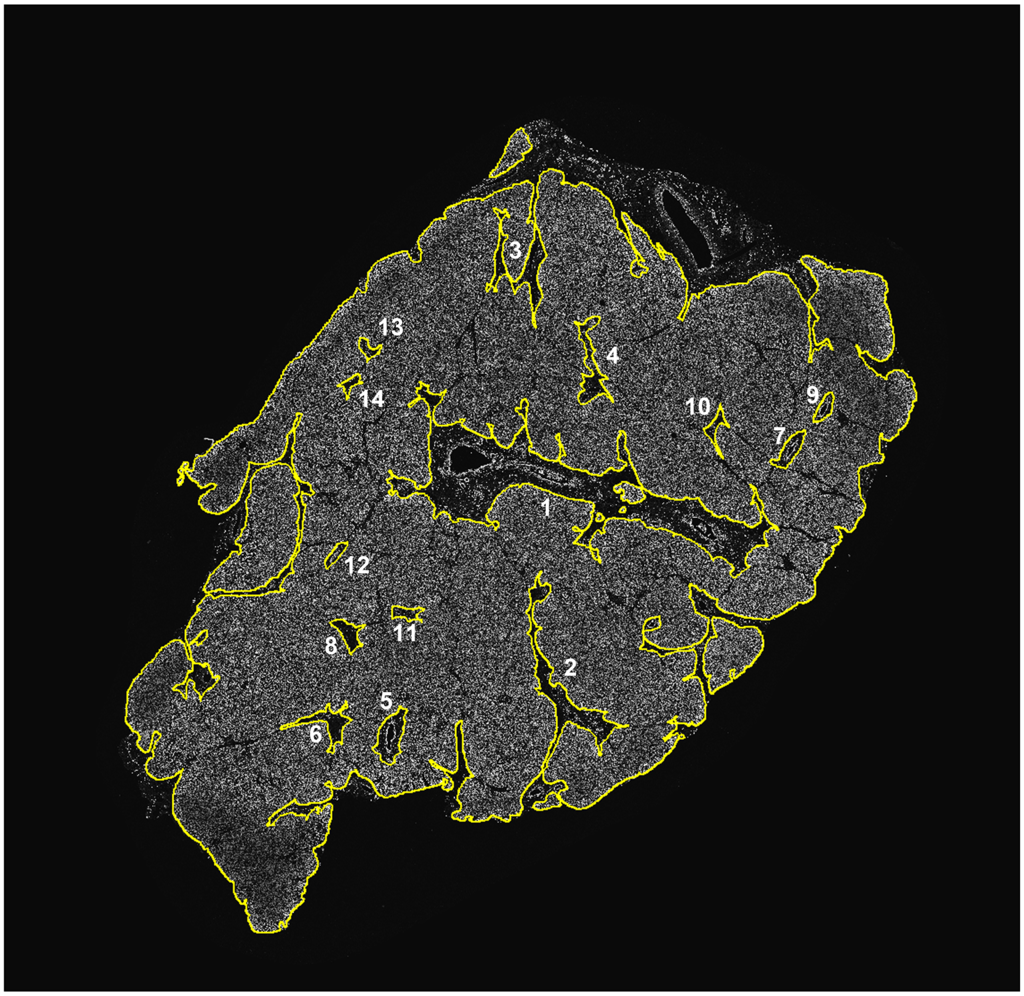

Total area = 64.072(mm^2)

#	Area (mm^2)	%
1	3.479	5.43
2	0.777	1.21
3	0.367	0.57
4	0.207	0.32
5	0.202	0.31
6	0.159	0.25
7	0.102	0.16
8	0.094	0.15
9	0.062	0.10
10	0.061	0.09
11	0.057	0.09
12	0.046	0.07
13	0.043	0.07
14	0.038	0.06
**Total**	5.692	8.88

S2. Contour Area Macro.

```
//Fiji/ImageJ macro returns the area of the contour/pancreas area in um^2
//The contour selection should be added to the ROI manager and selected
run("Select None");
width = getWidth;
height = getHeight;
newImage("mask", "8-bit White", width, height, 1);
total=roiManager("count");
for (n=0;n<total;n++){
        roiManager("select",n);
        setColor(0, 0, 0);
        fill();
}
//set scale and measure selection area scale. Note Scale is variable depending
on the microscope and lens used, in this example a scale of 0.645 (pixels/
um^2) is used
run("Set Measurements...", "area limit redirect=None decimal=3");
run("Properties...", "channels=1 slices=1 frames=1 unit=micron pixel_width=.
645 pixel_height=.645 voxel_depth=.645 frame=[0 sec] origin=0,0");
run("Select None");
setAutoThreshold("Default");
run("Create Selection");
run("Measure");
```

S3. Image Processing Macro.

```
//Fiji/ImageJ macro takes four images, each of a different channel for the
same sample (SST, INS, DAPI, GLU)
//Assumes the channel names are somewhere in the filename for each image
if (nImages() < 4) {
        //macro requires at least four images.
        waitForUser("This macro requires at least four single-channel
images.");
        exit();
}
//Note Scale is variable depending on the microscope and lens used, in this
example a scale of 0.645 (pixels/um^2) is used
image_titles = newArray(nImages());
for(image_i=1; image_i <= nImages(); image_i++) {
        selectImage(image_i);
        image_name=getTitle();
        if (indexOf(image_name, "SST") >= 0) {
                image_titles[0] = image_name;
                curr_dir = getDirectory("image");
                curr_name = File.getName(curr_dir);
                print(curr_name + " open");
        } else if (indexOf(image_name, "INS") >= 0) {
                image_titles[1] = image_name;
        } else if (indexOf(image_name, "DAPI") >= 0) {
                image_titles[2] = image_name;
        } else if (indexOf(image_name, "GLU") >= 0) {
                image_titles[3] = image_name;
        }
        run("Properties...", "unit=um pixel_width=0.645 pixel_height=0.645
voxel_depth=0 origin=0,0");
}
        if (!File.exists(curr_dir+"/Final/"))
                File.makeDirectory(curr_dir+"/Final/");
        if (!File.exists(curr_dir+"/Final/"))
                exit("Unable to create directory");
//select each channel and allow user to set threshold value, save threshold
values in 4channelstoROIs_paramters.txt
//and save images in Final folder
image_thresholds = newArray(nImages());
for(slice_i=0; slice_i < image_titles.length; slice_i++) {
        if (image_titles[slice_i] != "") {
                selectWindow(image_titles[slice_i]);
                if (slice_i==0) {
                        channel="SST";
```

```
		} else if (slice_i==1) {

			channel="INS";
		} else if (slice_i==2) {

			channel="DAPI";
		} else if (slice_i==3) {

			channel="GLU";
		}

	if (!File.exists(curr_dir+"/Final/"+curr_name+channel+".Final.tif")){
		save(curr_dir+"/Final/"+curr_name+channel+".Final.tif");
	}
		run("8-bit");
		rename(channel);
		run("Threshold...");

		waitForUser("Pick a representative threshold value for " +
channel + "\nPress 'OK' to input");

		getThreshold(low,high);
		File.append(channel+" threshold: "+low+","+high,curr_dir
+"4channelstoROIs_parameters.txt");

	}
}
```

S4. Image Analysis Macro.

```
//This script uses linux bash shell commands and and imageJ functions
//ImageJ and the script can be run by passing java options the on linux
command line. Setting an alias for the operation makes it easier to use
//The alias "imageAnalysis" is used here. Operation logs from the script are
saved at/var/log/imageAnalysis/jobs.log
//"imageAnalysis" takes in 3 input parameters.
//First parameter is the directory path to the folder which contains the
"Final" folder and a .txt file with the threshold values for 4 channels.
"Final" folder should contain a set of 3 cytoplasmic marker stained channel
images and 1 nuclear marker channel image for example INS.tif,GLU.tif,SST.tif
and DAPI.tif
//The second parameter allows users to set the scale of images (pixels/um^2)
as the scale can vary depending on the microscope and lens used
//The third parameter allows users to set the number of pixels required for
expanding/contracting selection obtained from the 3 channels stained for
cytoplasmic marker to obtain islet selection.
//Batchmode for faster analysis
setBatchMode(true);
//Split input argument and check for 3 input parameters
parameters=split(getArgument(),",");

if (lengthOf(parameters)<1) dir="Null"; else dir=parameters[0];
if (lengthOf(parameters)<2) scale="Null"; else scale=parameters[1];
if (lengthOf(parameters)<3) enlarge="Null"; else enlarge=parameters[2];
//Exit if any paramteres are missing and save message in the log file
if(lengthOf(parameters)!=3){
        getDateAndTime(year, month, dayOfWeek, dayOfMonth, hour, minute,
second, msec);
        if(File.exists("/var/log/imageAnalysis/jobs.log")){
                File.append(dayOfMonth +"/"+month+1 +"/"+year+" "+hour
+":"+minute+">> ERROR: Parameters missing. Exiting. (Given dir="+dir+",
scale="+scale+", enlarge="+enlarge+").","/var/log/imageAnalysis/jobs.log");
        }
     run("Quit");
}

//get file info and save info to log file
getDateAndTime(year, month, dayOfWeek, dayOfMonth, hour, minute, second,
msec);
print(dayOfMonth +"/"+month+1 +"/"+year);
starttime=getTime();
if(File.exists("/var/log/imageAnalysis/jobs.log")){
                File.append(dayOfMonth +"/"+month+1 +"/"+year+" "+hour
+":"+minute+">> Starting imageAnalysis on "+dir+" with scale="+scale+",
```

```
enlarge="+enlarge, "/var/log/imageAnalysis/jobs.log");
}
run("Input/Output...", "jpeg=75 gif=-1 file=.txt copy_row save_column
save_row");
//Get the image directory and check for file containing threshold values
"imageAnalysisnelstoROIs_parameters.txt"
curr_dir=dir;
curr_name = File.getName(curr_dir);
if(File.exists(curr_dir+"Final/"+curr_name+".dapi.coords.txt")){
                print(File.rename(curr_dir+"Final/"+curr_name
+".dapi.coords.txt", curr_dir+"Final/"+curr_name+".dapi.coords.txt."+random
+".bak"));
}
if(File.exists(dir+"imageAnalysisnelstoROIs_parameters.txt")) {
                filestring=File.openAsString(dir
+"imageAnalysisnelstoROIs_parameters.txt");
        }
else {
                if(File.exists("/var/log/imageAnalysis/jobs.log")){
                        File.append(dayOfMonth +"/"+month+1 +"/"+year+" "+hour
+":"+minute+">> ERROR: "+dir+" contains no parameters", "/var/log/
imageAnalysis/jobs.log");
                }
        run("Quit");
}
//Check if all 4 channel images i.e. 3 cytoplamic (INS,GLU & SST) stained  and
1 nuclear stained (DAPI) channels are present in the "Final" folder in the
image directory
list=getFileList(dir+"/Final/");
comp=0;
        for (i=0; i<list.length; i++) {
        if (indexOf(list[i], "INS") != -1){
                fnINS=list[i];
                        comp++;
                        print(fnINS);
                }

        else if (indexOf(list[i], "SST") != -1) {
                        print(list[i]);
                        fnSST=list[i];
                        comp++;
                }
        else if (indexOf(list[i], "GLU") != -1) {
                        print(list[i]);
                        fnGLU=list[i];
                        comp++;
                }
```

```
                else if (indexOf(list[i], "DAPI") != -1) {
                        print(list[i]);
                        fnDAPI=list[i];
                        comp++;
                }

    }

if (comp <4) {
        if(File.exists("/var/log/imageAnalysis/jobs.log")){
                File.append(dayOfMonth +"/"+month+1 +"/"+year+" "+hour
+":"+minute+">> ERROR: "+dir+"/Final/ does not contain INS, SST, GLU, and DAPI
files", "/var/log/imageAnalysis/jobs.log");
        }
        run("Quit");
}

rows=split(filestring, "\n");
x=newArray(rows.length);
y=newArray(rows.length);

if(rows.length <4){
    if(File.exists("/var/log/imageAnalysis/jobs.log")){
                File.append(dayOfMonth +"/"+month+1 +"/"+year+" "+hour
+":"+minute+">> ERROR: "+dir+" Less than 4 parameters were found", "/var/log/
imageAnalysis/jobs.log");
        }
        run("Quit");
}
//get threshold values from "imageAnalysisnelstoROIs_parameters.txt" file
comp=0;
for(i=rows.length-4; i<rows.length; i++){
                columns=split(rows[i],": ,");
        if (indexOf(columns[0], "INS") != -1){
           tholdINS=columns[2];
                        comp++;
                }
        else if (indexOf(columns[0], "SST") != -1) {
            tholdSST=columns[2];
                        comp++;
                }
        else if (indexOf(columns[0], "GLU") != -1) {
            tholdGLU=columns[2];
                        comp++;
                }
                else if (indexOf(columns[0], "DAPI") != -1) {
                        tholdDAPI=columns[2];
```

```
                        comp++;
                }
}

if (comp <4) {
        if(File.exists("/var/log/imageAnalysis/jobs.log")){
                File.append(dayOfMonth +"/"+month+1 +"/"+year+" "+hour
+":"+minute+">> ERROR: "+dir+"/Final/bbb does not contain INS, SST, GLU, and
DAPI files", "/var/log/imageAnalysis/jobs.log");
        }
        run("Quit");
        }

//set thresholds for all 4 channels and covert to masks
print("Opening: "+dir + "Final/" + fnINS);
open(dir + "Final/" + fnINS);
rename("INS");
run("8-bit");
setThreshold(tholdINS,255);
run("Convert to Mask");
rename("INS_mask");

open(dir + "/Final/" + fnSST);
rename("SST");
run("8-bit");
setThreshold(tholdSST,255);
run("Convert to Mask");
rename("SST_mask");

open(dir + "/Final/" + fnGLU);
rename("GLU");
run("8-bit");
setThreshold(tholdGLU,255);
run("Convert to Mask");
rename("GLU_mask");

open(dir + "/Final/" + fnDAPI);
rename("DAPI");
run("8-bit");
run("Smooth");
setThreshold(tholdDAPI,255);
run("Convert to Mask");
rename("DAPI_mask");

print(curr_name + " open");

//set scale
```

```
selectWindow("SST_mask");
run("Properties...", "unit=um pixel_width="+scale+" pixel_height="+scale+"
voxel_depth=0 origin=0,0 global");

//Combine mask area from the 3 hormone channels i.e. SST, INS, GLU
imageCalculator("Add create", "SST_mask","INS_mask");
showProgress(6/9);

imageCalculator("Add create", "Result of SST_mask","GLU_mask");
showProgress(7/9);

//set measurements, run analyze particles, summarize results on the combined
image and save image as composite-mask.tif
selectWindow("Result of Result of SST_mask");
run("Set Measurements...", "area centroid center perimeter bounding shape
feret's limit redirect=None decimal=3");
setAutoThreshold("Default dark");
setThreshold(20, 255);
showProgress(8/9);
setAutoThreshold("Default dark");
setThreshold(20, 255);
run("Analyze Particles...", "size=170-Infinity circularity=0.00-1.00
show=Nothing clear summarize");
save(curr_dir+curr_name+".Composite-mask.tif");
rename("merged");

//select islet areas from the combined image, set threshold and get islet
measurements
setAutoThreshold("Default dark");
setThreshold(20, 255);
run("Create Selection");
run("Enlarge...","enlarge="+enlarge);
run("Enlarge...","enlarge=-"+enlarge);
run("Create Mask");
setAutoThreshold("Default dark");
setThreshold(20, 255);
run("Analyze Particles...", "size=170-Infinity circularity=0.00-1.00
show=Outlines include display clear summarize add");
save(curr_dir+curr_name+".Composite-outlines-masks.tif");

close();
showProgress(1);

// get measurements for 3 cytoplasmic channels within islets for all islets in
the section and set additional measurement parameters
selectWindow("merged");
n = roiManager("count");
```

```
print(n);
showProgress(0);
for (i = 0; i <= n-1; i++) {
        roiManager("Select", i);
        total_area = getResult("Area",i);

        selectWindow("SST_mask");
        roiManager("Measure");
        SST_area= getResult("Area",n+(3*i));
        selectWindow("INS_mask");
        roiManager("Measure");
        INS_area= getResult("Area",n+(3*i+1));
        selectWindow("GLU_mask");
        roiManager("Measure");
        GLU_area= getResult("Area",n+(3*i)+2);

        fluo_area=SST_area+GLU_area+INS_area;
        setResult("SST Area",i,SST_area);
        setResult("SST Percent Area",i,SST_area/fluo_area);
        setResult("INS Area",i,INS_area);
        setResult("INS Percent Area",i,INS_area/fluo_area);
        setResult("GLU Area",i,GLU_area);
        setResult("GLU Percent Area",i,GLU_area/fluo_area);
        setResult("Fluorescent Area",i,fluo_area);
        updateResults();
}
IJ.deleteRows(n,nResults);
saveAs("Measurements", curr_dir+"Final/"+curr_name+".results.xls");

//duplicate image mask for all channels for downstream analysis
for (q = 0; q <= n-1; q++) {
        selectWindow("SST_mask");
        roiManager("Select", q);
        run("Duplicate...", "title=SST_ROI_"+q);
        run("Duplicate...", "title=SST_ROI_DAPI_"+q);

        selectWindow("INS_mask");
        roiManager("Select", q);
        run("Duplicate...", "title=INS_ROI_"+q);
        run("Duplicate...", "title=INS_ROI_DAPI_"+q);

        selectWindow("GLU_mask");
        roiManager("Select", q);
        run("Duplicate...", "title=GLU_ROI_"+q);
        run("Duplicate...", "title=GLU_ROI_DAPI_"+q);

        selectWindow("DAPI_mask");
```

```
        roiManager("Select", q);
        run("Duplicate...", "title=DAPI_ROI_"+q);

        //Run watershed on the Dapi channel, set measurements and run analyze
particles
        selectWindow("DAPI_ROI_"+q);
        run("Watershed");
        run("Set Measurements...", "area centroid limit redirect=None
decimal=3");
        run("Analyze Particles...", "size=20-Infinity circularity=0.00-1.00
show=Nothing display summarize add");

        //For each dapi ROI expand, find which channel dapi nuclei belongs to
and count each cell type
        showProgress(q/n);
        IJ.deleteRows(n,nResults);
        SST_cells = 0;
        INS_cells = 0;
        GLU_cells = 0;
        SST_islet_area=0;
        INS_islet_area=0;
        GLU_islet_area=0;
        print(roiManager("count")-n);
        for (j = 0; j<roiManager("count")-n; j++) {
                selectWindow("DAPI_ROI_"+q);
                roiManager("Select", j+n);
                setThreshold(0,255);
                roiManager("Measure");
                dapi_area= getResult("Area",n+(j*4));
                dapi_x= getResult("X",n+(j*4));
                dapi_y= getResult("Y",n+(j*4));

                run("Enlarge...", "enlarge=1");

                selectWindow("SST_ROI_"+q);
                roiManager("Select", j+n);
                setThreshold(1,255);
                roiManager("Measure");
                SST_area= getResult("Area",n+(j*4)+1);

                selectWindow("INS_ROI_"+q);
                roiManager("Select", j+n);
                setThreshold(1,255);
                roiManager("Measure");
                INS_area= getResult("Area",n+(j*4)+2);

                selectWindow("GLU_ROI_"+q);
```

```
			roiManager("Select", j+n);
			setThreshold(1,255);
			roiManager("Measure");
			GLU_area= getResult("Area",n+(j*4)+3);

			max_area = maxOf(maxOf(SST_area, INS_area),GLU_area);

			if (max_area > 0) {
				if (max_area == SST_area) {
					File.append(q+1+" "+j+1+" "+dapi_x+" "+dapi_y
+" 1 "+getResult("Area",q), curr_dir+"Final/"+curr_name+".dapi.coords.txt");
					selectWindow("SST_ROI_DAPI_"+q);
					roiManager("Select", j+n);
					setBackgroundColor(0, 0, 0);
					run("Clear");
					SST_cells++;
				}
				else if (max_area == INS_area) {
					File.append(q+1+" "+j+1+" "+dapi_x+" "+dapi_y
+" 2 "+getResult("Area",q), curr_dir+"Final/"+curr_name+".dapi.coords.txt");
					selectWindow("INS_ROI_DAPI_"+q);
					roiManager("Select", j+n);
					setBackgroundColor(0, 0, 0);
					run("Clear");
					INS_cells++;
				}
				else if (max_area == GLU_area) {
					File.append(q+1+" "+j+1+" "+dapi_x+" "+dapi_y
+" 3 "+getResult("Area",q), curr_dir+"Final/"+curr_name+".dapi.coords.txt");
					selectWindow("GLU_ROI_DAPI_"+q);
					roiManager("Select", j+n);
					setBackgroundColor(0, 0, 0);
					run("Clear");
					GLU_cells++;
				}
				else {}

			}

		}

		while(roiManager("count")>n) {
			roiManager("Select", n);
			roiManager("Delete");
		}
		//multiply number of nuclei by the average nuclear area of 38 µm^2
		SST_dapi_area=SST_cells*38;
```

```
        INS_dapi_area=INS_cells*38;
        GLU_dapi_area=GLU_cells*38;
        total_dapi_area=SST_dapi_area+INS_dapi_area+GLU_dapi_area;
        IJ.deleteRows(n,nResults);
        //Set results parameters
        setResult("INS_Cells",q,INS_cells);
        setResult("INS_dapi_Area",q,INS_dapi_area);
        updateResults();
        setResult("SST_Cells",q,SST_cells);
        setResult("SST_dapi_Area",q,SST_dapi_area);
        updateResults();
        setResult("GLU_Cells",q,GLU_cells);
        setResult("GLU_dapi_Area",q,GLU_dapi_area);
        updateResults();
        setResult("Total_DAPI_Area",q,total_dapi_area);
        updateResults();
        selectWindow("INS_ROI_DAPI_"+q);
        close();
        selectWindow("SST_ROI_DAPI_"+q);
        close();
        selectWindow("GLU_ROI_DAPI_"+q);
        close();
        selectWindow("INS_ROI_"+q);
        close();
        selectWindow("SST_ROI_"+q);
        close();
        selectWindow("GLU_ROI_"+q);
        close();
        selectWindow("DAPI_ROI_"+q);
        close();

}

selectWindow("SST_mask");
close();
selectWindow("INS_mask");
close();
selectWindow("GLU_mask");
close();

//save ROI measurements.
saveAs("Measurements", curr_dir+"Final/"+curr_name+".cells.results.xls");
saveAs("Measurements", curr_dir+"Final/"+curr_name+".cells.results.txt");
//close all open images
while (nImages()>0) {
        selectImage(nImages());
        run("Close");
```

```
}
//add details of analysis completion to the log file
getDateAndTime(year, month, dayOfWeek, dayOfMonth, hour, minute, second,
msec);
if(File.exists("/var/log/imageAnalysis1/jobs.log")){
        File.append(dayOfMonth +"/"+month+1 +"/"+year+" "+hour+":"+minute+">>
Successfully finished imageAnalysis on "+dir+" after "+round((getTime()-
starttime)/60000)+" minutes", "/var/log/imageAnalysis1/jobs.log");
}
run("Quit");
```

S5. Macro for Islet Size Distribution and Cellular Composition.

```
%Matlab script generates figure for cellular composition in relation to islet
size distribution as shown in Fig 6A.
%import excel spreadsheet obtained from the Image Ananlysis macro
"[filename].results.xls" as a tab delimited file and starting at column 1 and
row 1  put all data into matrixA, change columns if otherwise
clear all;
matrixA=dlmread(['FilePath\FileName.results.xls'],'\t',1,1);
arrayIslets=(matrixA(:,26)); % put islet areas into a array
arraySST= (matrixA(:,20));% " put somatostatin channel areas into an array "
arrayINS= (matrixA(:,22));% " put Insulin channel areas into an array "
arrayGLU= (matrixA(:,24));% " put glucagon channel areas into an array  "
matrix1= cat(2, arrayIslets, arraySST, arrayINS,  arrayGLU); % put all columns
into matrix
matrix=matrix1;
matrix(isnan(matrix))=0; % Convert all NAN values to Zero
area= log2(matrix(:,1)/178); % find which bin each islet falls into
area(find(area <0))=0;       % any bin less than 0 should be in the 0 bin
areaAdjust= floor(area);     % floor all log2 values into intergers
matrix(:,6)= areaAdjust;    % make this the last column
clear area areaAdjust        % clean up
totals= [sum(matrix(:,2)), sum(matrix(:,3)), sum(matrix(:,4))];
% for each bin size in the data set (up to max), iterate through each binsize
for i=1:max(matrix(:,6));
                % extract the rows that match the binsize i
        relevant= matrix(matrix(:,6) == i,:);
        % calculate the SST percent area of each islet
                SST= relevant(:,2)./relevant(:,5);
        SST_area= SST;
        SST_area(isnan(SST_area))=0; % get rid of NANS
        % mean SST percent in this binsize
                SST_area_mean(i)= mean(SST_area);
                ; % SEM of SST% in this binsize
                SST_area_sem(i)= std(SST_area)./sqrt(length(SST_area))
                % calculate the INS% area of each islet
                INS= relevant(:,3)./relevant(:,5);
                INS_area= INS;
        INS_area(isnan(INS_area))=0; % get rid of NANS
                % mean INS% in this binsize
                INS_area_mean(i)= mean(INS_area);
                % SEM of INS percent in this binsize
                INS_area_sem(i)= std(INS_area)./sqrt(length(INS_area));
        % calculate the GLU percent area of each islet
                GLU= relevant(:,4)./relevant(:,5);
        GLU_area= GLU;
```

```
        GLU_area(isnan(GLU_area))=0; % get rid of NANS
        % mean GLU percent in this binsize
                GLU_area_mean(i)= mean(GLU_area);
        % SEM of GLU percent in this binsize
                GLU_area_sem(i)= std(GLU_area)./sqrt(length(GLU_area));
        % number of islets in this binsize
                count(i)= size(relevant,1);
end
countpercent= count/ size(matrix,1); % what percent of total islets were in
each binsize?
figure; % generate figure box
set(gca,'LooseInset',get(gca,'TightInset')); % smallest margins possible:
http://undocumentedmatlab.com/blog/axes-looseinset-property/
set(gca,'fontsize', 15,'fontname', 'Arial','fontweight','bold'); % setting
axis font color and weight
areaplotx= 0:length(countpercent)-1; % indexing started at 1, knock it down to
0 again
bar(areaplotx, countpercent, 'facecolor',[.75 .75 .75]); % plot bar graph of
%islets in each bin
hold on; % wait for more to be plotted
% plot line for each hormone measured
errorbar(areaplotx, GLU_area_mean, GLU_area_sem,'r','linewidth',2);
errorbar(areaplotx, INS_area_mean, INS_area_sem,'g','linewidth',2);
errorbar(areaplotx, SST_area_mean, SST_area_sem,'b','linewidth',2);
% set y axis limit at 100%, set x axis to allow for whole bar to be shown
ylim([0 1]); xlim([-.5 length(countpercent)-.5]);
% clean up and done
clear count countpercent matrix i relevant areaplotx ...
    SST_area SST_area_mean SST_area_sem...
    INS_area INS_area_mean INS_area_sem...
    GLU_area GLU_area_mean GLU_area_sem matrix
```

S6. Macro for Islet Size Contribution.

```
% Matlab script generates figure for contribution of islet sizes for total
islet area in Fig 6B.
% import excel spreadsheet obtained from the Image Ananlysis macro
"[filename].results.xls" as an excel file, change columns if otherwise
clear all;
matrixA= xlsread(['FilePath\FileName.results.xls']);
arrayIslets=(matrixA(:,27)); % put islet areas into an array
arraySST= (matrixA(:,21));% put somatostatin areas into an array
arrayINS= (matrixA(:,23));% put insulin areas into an array
arrayGLU= (matrixA(:,25));% put glucagon areas into an array
% put all columns into matrix
matrix1= cat(2, arrayIslets, arraySST, arrayINS,  arrayGLU);
matrix=matrix1;
% Convert all NAN values to Zero
matrix(isnan(matrix))=0;
% find which bin each islet falls into
area= log2(matrix(:,1)/178);
% any bin less than 0 should be in the 0 bin
area(find(area <0))=0;
% floor all log2 values into intergers
areaAdjust= floor(area);
% make this the last column
matrix(:,6)= areaAdjust;
% cleanup
clear area areaAdjust

totals= [sum(matrix(:,2)), sum(matrix(:,3)), sum(matrix(:,4))];

% for each bin size in the data set (up to max), iterate through each binsize
for i=1:max(matrix(:,6));
                % extract the rows that match the binsize i
        relevant= matrix(matrix(:,6) == i,:);
        summed_area(i)= sum(relevant(:,2));
        sem_summed_area(i)= std(relevant(:,2))./ sqrt(length(relevant(:,2)));
        % number of islets in this binsize
                count(i)= size(relevant,1);

end

total= sum(matrix(:,2));
% what percent of total islets were in each binsize?
countpercent= count/ size(matrix,1);
figure; % generate figure box
% smallest margins possible: http://undocumentedmatlab.com/blog/axes-
```

```
looseinset-property/
set(gca,'LooseInset',get(gca,'TightInset'));
% setting axis font color and weight
set(gca,'fontsize', 15,'fontname', 'Arial','fontweight','bold');
% indexing started at 1, knock it down to 0 again
areaplotx= 0:length(countpercent)-1;
% plot bar graph of percent sets in each bin
bar(areaplotx, countpercent, 'facecolor',[.75 .75 .75]);
hold on; % wait for more to be plotted
% plot error bars
errorbar(areaplotx, summed_area/total, sem_summed_area/total ,'r','linewidth',
2,'markersize',20);
% set y axis limit at 35%, set x axis to allow for whole bar to be shown
ylim([0 0.35]); xlim([-.5 length(countpercent)-.5]);
```

S7. Macro for 3D Scatter Plots.

```
% Matlab script generates 3D scatter plot which shows relationship between
islet's Feret's diameter and circularity in relation to islet size
distribution as shown in Fig 6C.
% Density of the islets are shown as heatmap with blue and orange representing
dense and sparse islets
% import [filename].results.xls as an excel file, change columns if otherwise
clear all;
matrixA= xlsread(['FilePath\FileName.results.xls']);
% divide islet area by single cell area of 178 micro meter squared and get
log2 values
area= log2(matrixA(:,27)/178);
area(find(area <0))=0;
% floor all log2 values into intergers
areaAdjust= floor(area);
% extract area, feret diameter and circularity values to array
feret= matrixA(:,13);
circ= matrixA(:,12);
new= [area, feret, circ];
% specify HSV heatmap color
x= colormap('HSV');
dx= sum(squareform(pdist(new)) <.8, 2);
c= x(ceil((dx/ max(dx))*64),:);
figure;  %generate figure box
set(gca,'LooseInset',get(gca,'TightInset')); % smallest margins possible
stem3(area, circ, feret,'Color',[84/255 84/255 84/255], 'Marker', 'none',
'Linewidth', 1); hold on;
% specify axes limits and tick marks
xlim([0 11]);  set(gca, 'Xtick',[0:1:11]); set(gca, 'Ytick',[0:.2:1]); zlim([0
600]);
% setting axis font color and weight
set(gca,'fontsize', 16,'fontname', 'Arial','fontweight','bold');box off;
hold on;
scatter3(area, circ, feret, 20, c, 'filled'); % plot 3D scatter
clear area areaAdjust circ color feret matrix;
```

Table S1: Results of simulated analysis of each individual case

**Case**	**A**	**B**	**C**	**D**	**E**	**F**	**G**	**H**	**I**	**J**
Sex	F	M	M	F	F	F	F	M	M	F
Age (yr)	20	44	50	51	80	26	30	47	59	66
BMI	23.1	36	23	21.1	24.6	40	47.4	30	21.2	31
T2D (duration)	-	-	-	-	-	6 mo	3 mo	4 yr	14 yr	3 yr
Number of total blocks	36	38	62	42	38	64	42	64	34	54
Number of blocks used (paraffin embedded)	18	19	31	21	19	32	21	32	17	27
Endocrine cell mass(%; whole pancreas)	1.30 (± 0.09)	2.00 (± 0.19)	0.42 (± 0.07)	2.11 (± 0.13)	1.14 (± 0.13)	1.97 (± 0.17)	1.99 (± 0.25)	0.79 (± 0.07)	1.79 (± 0.28)	0.70 (± 0.08)
**Simulation**	**Range of fold differences from the whole pancreas analysis**									
1 block each from 3 regions (i.e. 3 blocks)	0.76-1.52	0.37-1.01	0.38-0.96	0.60-1.13	0.32-1.82	0.25-1.16	0.30-0.98	0.38-1.01	0.50-1.08	0.31-1.18
1 block from Head	0.83-1.19	0.57-1.51	0.80-1.34	0.84-1.19	0.27-1.44	0.48-1.72	0.69-1.63	0.54-1.34	0.76-1.21	0.49-1.86
1 block from Body	0.84-1.33	0.59-1.37	0.57-1.56	0.64-1.23	0.53-1.46	0.31-1.65	0.31-1.83	0.55-1.50	0.67-1.64	0.52-1.62
1 block from Tail	0.72-1.97	0.70-1.37	0.46-3.58	0.69-1.35	0.56-1.23	0.60-1.55	0.55-2.01	0.59-2.01	0.51-1.95	0.63-1.69
1 block from whole pancreas	0.61-1.52	0.37-1.80	0.38-5.38	0.51-1.63	0.29-1.82	0.24-2.22	0.29-2.62	0.96-2.90	0.40-2.81	0.31-2.63
